# Renal Morphology in Coronavirus Disease: A Literature Review

**DOI:** 10.3390/medicina57030258

**Published:** 2021-03-11

**Authors:** Patrick de Oliveira, Kaile Cunha, Precil Neves, Monique Muniz, Giuseppe Gatto, Natalino Salgado Filho, Felipe Guedes, Gyl Silva

**Affiliations:** 1 Nephrology Service, Onofre Lopes University Hospital, Federal University of Rio Grande do Norte, Natal 59066-230, RN, Brazil; pkvanttinny@gmail.com (P.d.O.); felipeguedeshuol@gmail.com (F.G.); 2 University Hospital of Federal University of Maranhão, São Luis 65020-070, MA, Brazil; kac-doctor@uol.com.br (K.C.); moniqueprmuniz@gmail.com (M.M.); natalinosalgadofilho@uol.com.br (N.S.F.); 3 Nephrology Division, University of São Paulo Medical School, São Paulo 05403-000, SP, Brazil; precilmed61@yahoo.com.br; 4 Nephrology Service, University Hospital of Brasília, Brasília 70840-901, DF, Brazil; giuseppe.gatto@ebserh.gov.br; 5 Patology Department, Ribeirão Preto Medical School, University of São Paulo, Ribeirão Preto 14049-900, SP, Brazil

**Keywords:** renal morphology, coronavirus, renal biopsy, COVID-19

## Abstract

Renal biopsy is useful to better understand the histological pattern of a lesion (glomerular, tubulointerstitial, and vascular) and the pathogenesis that leads to kidney failure. The potential impact of severe acute respiratory syndrome coronavirus 2 (SARS-CoV-2) on the kidneys is still undetermined, and a variety of lesions are seen in the kidney tissue of coronavirus disease patients. This review is based on the morphological findings of patients described in case reports and a series of published cases. A search was conducted on MEDLINE and PubMed of case reports and case series of lesions in the presence of non-critical infection by SARS-CoV-2 published until 15/09/2020. We highlight the potential of the virus directly influencing the damage or the innate and adaptive immune response activating cytokine and procoagulant cascades, in addition to the genetic component triggering glomerular diseases, mainly collapsing focal segmental glomerulosclerosis, tubulointerstitial, and even vascular diseases. Kidney lesions caused by SARS-CoV-2 are frequent and have an impact on morbidity and mortality; thus, studies are needed to assess the morphological kidney changes and their mechanisms and may help define their spectrum and immediate or long-term impact.

## 1. Introduction

The SARS-CoV-2, first described in humans in December 2019, in Wuhan, China, is the third coronavirus to appear in the last 20 years. The syndrome caused by this virus, COVID-19, has become a serious global public health emergency, causing a pandemic with an exponential increase in the number of people infected and deaths. Non-severe symptoms of COVID-19 infection such as fever, fatigue, anosmia, and cough progress in the first week, and dyspnea may arise 5 to 8 days after symptom onset. The main complication is ARDS requiring invasive mechanical ventilation in 12% to 24% of hospitalized patients and AKI in 37% of hospitalized patients. In a large prospective cohort of 701 patients with COVID-19 in China, 44% had proteinuria and 27% had hematuria on admission. During hospitalization, 5.1% developed AKI symptoms. On the other hand, in a retrospective Chinese cohort comprising 193 patients hospitalized for COVID-19, 59% had proteinuria, 44% hematuria, and 10% to 14% presented with azotemia. However, the potential impact of SARS-CoV-2 on the kidneys is still undetermined, and there are a variety of lesions in the renal tissue of patients in the presence of COVID-19. This review is based on the morphological kidney findings described in case reports and a series of published cases. A search was carried out on MEDLINE and PubMed of case reports and a series of cases of kidney lesions in the presence of non-critical infection by SARS-CoV-2 published until 15/09/2020.

## 2. Pathogenesis

SARS-CoV-2 is a beta-coronavirus that displays spike protein through which the viral RNA enters the host cell through its binding to the ACE-2. ACE-2 is highly expressed in several organs, mainly in type II alveolar epithelial cells. Subsequently, TMPRSS2 mediates the endocytosis of the virus; thus, the infected cells become unable to perform their functions. Following this, initially a specific adaptive immune response occurs in the early stages to eliminate the virus and control disease progression. Subsequently, a large endothelial inflammatory response arises with organ dysfunction, necrosis, and apoptosis of T cells and fulminant activation/consumption of coagulation factors.

## 3. Morphological Kidney Findings in COVID-19

In the kidney, ACE-2 is expressed in several cell types, including mesangial cells, podocytes, the parietal epithelium of Bowman’s capsule, and collecting ducts. The mechanisms of renal involvement in COVID-19 are still unclear, but there is a proposed multifactorial pathway: direct viral action and replication causing renal dysfunction; imbalance in the homeostasis of the renin-angiotensin-aldosterone system; deregulation of the complement system cascade; management of fluid therapy during the treatment of ARDS; procoagulant state; and consequence of a “cytokine storm” by the systemic inflammatory response.

**Figure 1:**
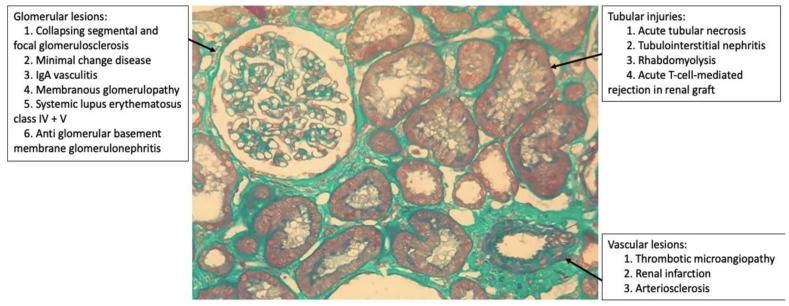
Histopathological patterns of renal lesions by SARS-CoV-2 (glomerular, tubulointerstitial, and vascular).

Renal biopsy is useful to better understand the histological pattern of the lesion (glomerular, tubulointerstitial, and vascular; Figure 1) and the pathogenesis that leads to AKI. However, due to the hemodynamic and ventilatory instability of patients as well as the use of anticoagulants that increase the risk of bleeding, this is a difficult task. In addition, it is not being performed in most hospitals because of the risk of exposure to staff, and often the procedure is not considered essential for the case.

Although acute tubular injury due to hemodynamic instability is probably the main factor for AKI in patients with severe COVID-19, the possibility of infection of the renal parenchyma directly or indirectly by the virus was suggested in patients with relatively mild respiratory symptoms, without septic shock or ARDS [1].

The first case series described was that of 26 autopsies of patients with COVID-19 who presented moderate to severe acute tubular necrosis and arteriosclerosis; the particles detected were suggestive of SARS-CoV-2 with electron microscopy in the tubular epithelium and podocytes [2]. However, some experts claim that these particles are only clathrin-coated vesicles, which are organelles of normal cells involved in intracellular transport [3]. In another series of autopsies, the viral RNA of SARS-CoV-2 in the kidney was quantified, especially in the glomeruli [4]. Finally, 3 out of 6 autopsies presented viral infection-associated syncytia in renal tissue [5]. These findings suggest a direct viral action responsible for renal dysfunction.

### 3.1. Glomerular Injury

The first live patient with COVID-19 who underwent a published renal biopsy was an African-American patient with previous comorbidities (stage IIIa chronic kidney disease, type 2 diabetes mellitus, systemic arterial hypertension), who did not develop ARDS, but developed AKI and needed dialysis [6]. The histopathological lesions observed resembled those of other forms of this cFSGS with capillary tuft collapse, hypertrophy and hyperplasia of the podocytes and parietal epithelial cells, and protein absorption droplets within the glomerular epithelium. No viral particles were identified by electron microscopy, and SARS-CoV-2 was not detected in renal tissue by immunohistochemistry or in situ hybridization, suggesting no direct viral action. This pattern was replicated in subsequent case reports [1,6,7,8,9,10,11,12,13], and only five of them displayed particles suggestive of SARS-CoV-2 or clathrin vesicles in podocytes on electron microscopy [14,15,16,17]. Biopsies were performed in specialized centers in Nottingham (UK) [16]; Paris (France) [7,10,12]; Lausanne (Switzerland) [17]; New York (NY) [1,8,9,14,15], New Orleans (LA) [6,13] and Chapel Hill (NC) [11] in the United States. (Table 1 and Table 2).

The reports and series of published cases with SARS-CoV-2 infection and collapsing glomerulopathy were all African-American, except one Indian [14]; of those who underwent genotyping, only three did not have high-risk APOL1 alleles and one was a transplanted patient, but the donor and recipient carried high-risk HLA alleles (DR4 and B44, respectively) [8,12].

The high-risk variants of APOL-1 (genotypes G1/G1, G2/G2, or G1/G2) are present in 10% to 15% of the African-American population and confer greater susceptibility to podocytopathy commonly manifesting in cFSGS when subjected, for example, to inflammatory diseases [SLE and hemophagocytic syndrome], drugs (pamidronate and interferon), and viral infections (arboviruses, HIV, parvovirus B19, cytomegalovirus, and Epstein–Barr virus). 

A common factor in these etiologies is the activation of interferon. The presence of endothelial tubuloreticular inclusions that have been described as ’interferon footprints’ are commonly identified by electron microscopy in cFSGS, including in some of the published case reports [1,6,8,10,13,16]. The precise mechanism of the interaction of APOL-1 with interferon signaling pathways is unclear. Nevertheless, it is suggested that the second-HIT caused by immunological dysregulation by COVID-19 is probably similar to that seen in other diseases associated with collapsing glomerulopathy [12,16].

The clinical and pathological similarity with HIVAN and the remarkable relationship between viral infection and high-risk APOL-1 genotype suggests the term COVAN to describe this entity. This type of nephropathy should be distinguished from most cases of AKI in COVID-19, which is characterized by acute tubular injury. Patients of African descent who have COVID-19, AKI, and nephrotic proteinuria in the absence of hemodynamic instability should be suspected. Clinical trials with anti-inflammatory and anti-cytokine therapy should prioritize this patient phenotype. Regarding the evolution of published cFSGS cases (Table 1), 63% (17/27) required kidney replacement therapy, 76.4% (13/17) of these continued on hemodialysis, and only 7.4% (2/27) died during the period when cases were recorded.

An Indian patient first presented steroid-resistant MCD that progressed to cFSGS weeks later, suggesting that non-African Americans and/or asymptomatic COVID-19 patients may have a more benign podocytopathy, at least initially [7,14]. However, there are reports of MCD in an African-American patient and high-risk APOL-1 genotype [8].

The inflammatory environment surrounding COVID-19 can also activate or exacerbate immunomediated diseases by a “trigger” in predisposed individuals. Examples include the emergence of IgA vasculitis (Henoch-Schonlein purpura), crescentic class IV+V transformation of longstanding preexisting class II SLE and development of acute T cell-mediated rejection in transplant patients [8].

Other glomerulopathies triggered in the presence of COVID-19 infection were those of a patient with anti-MBG glomerulonephritis and two with MG [8]. There are reports of influenza pneumonia or other insults preceding anti-GBM glomeurulonephritis; thus, COVID-19 may have similar interactions. The main target antigen in MG is PLA2R, which is also expressed in the respiratory tract, suggesting a potential source of antigen presentation to incite or potentiate anti-PLA2R autoimmune responses. It cannot be excluded that all such associations are purely coincidental in COVID-19.

No specific treatment has been investigated for the treatment of glomerulopathies associated with COVID-19. Vardhana et al. recommend parsimony when considering early immunosuppression in patients with signs of inflammatory hyperactivation and adaptive immune dysfunction by the disease, as there is a high risk of increased viral dissemination [18].

### 3.2. Tubular Injury

In patients with cFSGS, typical tubulointerstitial lesions such as microcystic tubular dilation and tubular lesions were also present [9]. Only one of the published cases had no report of acute tubular necrosis [7]. Isolated acute tubular injuries were identified in four native kidneys and two transplanted kidneys, including one with infarction [8].

The absence of persistent hemodynamic instability or severe pulmonary involvement suggests that tubular injury in a patient with COVID-19 is not predominantly ischemic. The hypotheses are direct viral toxicity in tubular cells that express ACE-2, cytokine-mediated tubular lesions, and heavy proteinuria contributing to tubular necrosis [16]. These mechanisms are also speculated to be an etiology of rhabdomyolysis that injures muscle tissue releasing heme-containing myoglobin pigment causing tubular necrosis; renal histology can reveal the formation of pigmented casts obstructing tubules. However, the etiology is probably multifactorial in a complex interaction of sepsis, hypoxia, hypotension, and exposure to nephrotoxic agents [8].

Some patients presented inflammatory cells and edema in the interstitium, that is, AIN of varying degrees. However, the administration of a wide variety of medications, many of which are nephrotoxic, due to the lack of strong evidence of COVID-19 treatment at the beginning of the pandemic, makes it difficult to associate AIN with SARS-CoV-2.

### 3.3. Vascular Injury

SARS-CoV-2 can directly infect endothelial cells, platelets, and megakaryocytes, inducing platelet damage, endotheliitis, and apoptosis, which triggers the recruitment of macrophages and granulocytes that synthesize pro-inflammatory cytokines. Inflammation progresses, aggravates microvascular and tissue damage, stimulates the extrinsic coagulation pathway, and inhibits fibrinolysis, which triggers a consumption coagulopathy enhanced by the production of antiphospholipid antibodies and by hypoxia, that induces platelet aggregation and a decrease in anticoagulant factors. This environment potentiates arterial and venous thrombotic events. There are reports of patients with COVID-19, one of them with kidney-pancreas transplantation, presenting acute renal failure of varying degrees with radiological signs of renal artery thrombosis in the presence of hemodynamic stability and treated with full anticoagulation with good response. As such, the American Society of Hematology states that it is reasonable to consider thromboprophylaxis in the population at risk.

There is the possibility of formation of microthrombi not detected by radiology, manifesting as clinical and histopathological thrombotic microangiopathies with visualization of generalized microthrombi in renal tissue. In a report, there was no disseminated intravascular coagulation and no history of abortions or thrombosis. A comprehensive complement system panel suggested deregulation of the alternative pathway.

In vitro, cholesterol present in the cell membrane and viral envelope contributes to the replication of SARS-CoV-2, which acts as a key component in viral entry. Furthermore, the evidence that inflammation is directly linked to the pathogenesis of atherosclerosis justifies the findings of arteriosclerosis of varying degrees in kidney histology.

## 4. Conclusions

This work includes a series of published cases on kidney injury in the presence of non-critical infection by COVID-19 and a review on the subject. We highlight the potential of the virus to directly influence the damage or innate and adaptive immune response activating cytokine and procoagulant cascades, in addition to the genetic component triggering glomerular diseases, mainly cFSGS, tubulointerstitial, and even vascular diseases. Kidney lesions caused by SARS-CoV-2 are frequent and have an impact on morbidity and mortality. Therefore, multicenter studies, such as the Brazilian consortium for the study of COVID-19-associated kidney diseases [19], are important to investigate morphological kidney changes and their mechanisms and may help to define their spectrum and their immediate or long-term impact.

## Figures and Tables

**Table 1:** Clinical, laboratory findings and follow-up information in published patients with collapsing segmental and focal glomerulosclerosis during COVID-19 infection.

Case	Sex	Age(y)	Race	Comorbidities	Alb (g/dL)	PTN (g)	Hm	Cr Baseline (mg/dL)	Cr at RB (mg/dL)	HD	Follow-Up	Ref
1	F	47	AA	HTN, DLP, OSAS, DM2, GERD	4.0	3.3	N	1.0	8.27	Y	HD	Sharma [1] ^1^
2	M	49	AA	HTN, CI, PAOD, arthritis	3.1	2.6	N	0.95	10.1	Y	HD	Sharma [1] ^2^
3	F	44	AA	HTN, DM2, CKD, DLP	2.5	25.0	Y	1.4	11.0	Y	HD	Larsen [6]
4	M	53	AA	HTN, CI	1.3	16.5	N	1.02	2.19	N	CT	Couturier [7] ^1^
5	M	53	AA	HTN, HBV	2.2	2.65	N	1.35	5.98	N	CT	Couturier [7] ^2^
6	M	46	AA	Obese, OSAS	3.1	5.8	N	1.1	12.5	Y	HD	Kudose [8] ^1^
7	M	62	AA	HTN, prostate carcinoma	3.1	12.1	N	2.0	10.7	N	CT	Kudose [8] ^2^
8	M	62	AA	HTN, prostate carcinoma, DM2	2.4	19.0	N	1.0	11.6	N	CT	Kudose [8] ^3^
9	M	57	AA	HTN, HCV	2.5	6.2	N	1.1	4.9	N	CT	Kudose [8] ^4^
10	M	61	AA	HTN, Obese	2.5	9.0	N	Normal	15.0	Y	HD	Kudose [8] ^5^
11	M	46	AA	Obese, OSAS	2.9	5.8	Y	1.1	19.9	Y	HD	Peleg [9]
12	M	79	AA	Stroke, MGUS, CKD III, HTN	2.9	11.4	N	N/A	2,5	Y	HD	Gailard [10]
13	F	28	AA	Asthma	1.6	2.0	N	0.99	>6.5	Y	CT	Magoon [11]^1^
14	M	56	AA	HTN, CKD	0.8	21.0	N	2.0	7.72	Y	CT	Magoon [11]^2^
15	M	29	AA	CKD (urinary schistosomiasis), kidney transplant, cell rejection	2.8	4.3	N	2.0	6.04	N	CT	Lazareth [12]
16	M	63	AA	N/A	2.1	12.7	N	1.3	4.9	Y	HD	Wu [13] ^1^
17	F	64	AA	N/A	2.4	4.6	N	1.5	4.2	N	CT	Wu [13] ^2^
18	F	65	AA	N/A	2.6	13.6	N	1.3	2.9	Y	HD Death	Wu [13] ^3^
19	M	44	AA	N/A	2.5	25.0	Y	1.4	11.4	Y	HD	Wu [13] ^4^
20	M	37	AA	N/A	3.0	N/A	N	1.0	9.0	Y	HD Death	Wu [13] ^5^
21	M	56	AA	N/A	2.9	3.6	Y	1.2	6.7	Y	CT	Wu [13] ^6^
22	M	71	AS	HTN, DM2, BPH	2.0	18.5	N	1.19	4.49	Y	HD	Gupta [14] ^1^
23	M	54	AA	HTN. DM2, former smoker	1.6	16.0	N	1.29	4.67	N	CT	Gupta [14] ^2^
24	M	56	AA	CI, cardiac transplant, CKD	N/A	7.4	N	1.86	7.78	N	CT	Kadosh [15]
25	M	54	AA	HTN, Obese, CKD	N/A	3.2	Y	1.41	13.6	Y	CT	Noble [16] ^1^
26	M	45	AA	DM2,Obese, CKD, HTN, kidney transplant	N/A	1.9	Y	3.2	14.05	Y	HD	Noble [16] ^2^
27	M	63	AA	HTN	2.3	5.0	N	1.2	8.4	N	CT	Kissling [17]

M: male; F: famale; y: years; AA: afroamerican; AS: asian; HTN: hypertension; DM2: Diabetes Mellitus type 2; CKD: chronic kidney disease; DLP: dyslipidemia; OSAS: obstructive sleep apnea syndrome; MGUS: monoclonal gammopathy of unknown significance; CI: cardiac insufficiency; HBV: hepatitis B virus; HCV: hepatitis C virus; GERD: gastroesophageal reflux disease; PAOD: peripheral arterial obstructive disease; BPH: benign prostatic hyperplasia; Alb: serum albumin; g/dL: gram per deciliter; PTN: proteinuria by urine spot, urine protein-creatinine ratio or 24-h urine protein; g: gram; Hm: hematuria; Cr: serum creatinine; mg/dL: milligram per deciliter; RB: renal biopsy; HD: hemodyalisis; CT: conservative treatment; Y: yes; N: No; N/A: not available; Ref: reference; Note 1…6: case number.

**Table 2:** Pathologic findings in published patients with collapsing segmental and focal glomerulosclerosis during COVID-19 infection.

Case	APOL1	ATI	TIN	IFTA	VS	TRI	VP	vRNA	Ref
1	G1/G2	Diffuse	N/A	Mild	N/A	Y	N	N	Sharma [1] ^1^
2	G1/G2	Diffuse	Mild	N/A	N/A	Y	N	N	Sharma [1] ^2^
3	G1/G1	Important	Present	Moderate	N/A	Y	N	N	Larsen [6]
4	G1/G1	N/A	N/A	Mild	N/A	N/A	N/A	N	Couturier [7] ^1^
5	G1/G2	Present	Present	Mild	Present	N/A	N/A	N	Couturier [7] ^2^
6	Note 1	Present	Focal	Mild	Mild	N/A	N	N	Kudose [8] ^1^
7	Note 1	Present	Focal	Moderate	Moderate to severe	Y	N	N	Kudose [8] ^2^
8	Note 1	Present	N/A	Moderate	Moderate	N	N	N	Kudose [8] ^3^
9	Note 1	Present	Focal	Severe	Mild	N/A	N/A	N	Kudose [8] ^4^
10	Note 1	Present	Focal	Severe	Mild to moderate	N	N	N	Kudose [8] ^5^
11	G1/G1	Severe	Mild to moderate	Mild	Mild to moderate	N	N	N	Peleg [9]
12	N/A	Present	N/A	N/A	N/A	Y	N	N	Gailard [10]
13	G1/G1	Moderate to severe	Diffuse	Mild	N/A	N	N	N/A	Magoon [11] ^1^
14	G1/G2	Moderate	Mild	Mild	Severe	N	N	N/A	Magoon [11] ^2^
15	Note 2	Severe	N/A	N/A	N/A	N/A	N/A	N	Lazareth [12]
16	G1/G1	Focal	N/A	Mild	N/A	Y	N	N	Wu [13] ^1^
17	G2/G2	Diffuse	N/A	Mild	N/A	N	N	N	Wu [13] ^2^
18	G1/G1	Diffuse	N/A	Mild to moderate	N/A	Y	N	N	Wu [13] ^3^
19	G1/G1	Diffuse	N/A	Moderate	N/A	Y	N	N	Wu [13] ^4^
20	G1/G2	Diffuse	N/A	Moderate	N/A	N	N	N	Wu [13] ^5^
21	G1/G1	Diffuse	N/A	Mild	N/A	N/A	N/A	N	Wu [13] ^6^
22	N/A	Moderate to severe	N/A	N/A	Severe	N	Y	N/A	Gupta [14] ^1^
23	N/A	Moderate	Mild	N/A	N/A	N	Y	N/A	Gupta [14] ^2^
24	N/A	Present	N/A	N/A	N/A	N	Y	N/A	Kadosh [15]
25	N/A	Severe	N/A	Mild	N/A	Y	Y	N/A	Noble [16] ^1^
26	N/A	Severe	Present	Mild	Present	N/A	N/A	N/A	Noble [16] ^2^
27	G1/G1	Present	N/A	N/A	N/A	N	Y	N	Kissling [17]

Note 1: In this case series, three of the five patients with collapsed glomerulosclerosis who consented to all genetic studies had high-risk APOL1 genotypes (two with G1/G1 and one with G1/G2); Note 2: The donor had an APOL1 G0/G2 genotype (low risk); the receptor genotype was G0/G0 (low risk), but both have high risk human leukocyte antigen (HLA) for collapsing glomerulosclerosis with HLA-DR4 in the donor and HLA-B44 in the recipient. ATI: acute tubular injury; TIN: tubulointerstitial nephritis; IFTA: interstitial fibrosis and tubular atrophy; VS: vascular sclerosis; TRI: tubuloreticular inclusion; VP: viral particle in electronic microscopy; vRNA: viral RNA situ hybridization; Y: yes; N: no; N/A: not available; Ref: reference; Note 1…6: case number.

## Abbreviations

ACE-2	Angiotensin-converter enzyme receptor 2
AKI	Acute kidney injury
AIN	Acute interstitial nephritis
APOL1 (G1/G2)	Apolipoprotein L1 (genotype 1 e 2)
ARDS	Acute respiratory distress syndrome
anti-GBM	Antiglomerular basement membrane glomerulonephritis
cFSGS	Collapsing focal segmental glomerulosclerosis
COVAN	COVID-19-associated nephropathy
COVID-19	Coronavirus disease 2019
HIV	Human immunodeficiency virus
HIVAN	HIV-associated nephropathy
HLA	Human leukocyte antigen
IgA	Immunoglobulin A
MCD	Minimal change disease
MG	Membranous glomerulopathy
PLA2R	Phospholipase A2 174 receptor
RNA	Ribonucleic acid
SARS-CoV-2	Severe acute respiratory syndrome coronavirus 2
SLE	Systemic lupus erythematosus
TMPRSS2	Transmembrane serine protease 2
